# Staging of COVID-19 disease; using selected laboratory profiles for prediction, prevention and management of severe SARS-CoV-2 infection in Africa-review

**DOI:** 10.4314/ahs.v23i1.2

**Published:** 2023-03

**Authors:** Hakim Sendagire, Steven Kiwuwa, Ali Dhamani, Roselyne Akugizibwe, Yasin Lwasa, Andrew Bukenya, Hussein Kafero Mukasa, Patrick Kakeeto, Ziadah Nankinga, Godfrey Bbosa, Juliet Babirye, Harriet Nankabirwa, Susan Nabadda

**Affiliations:** 1 College of Health Sciences, Makerere University, Kampala, Uganda; 2 Faculty of Health Sciences, Islamic University in Uganda; 3 Mengo Hospital, Kampala, Uganda; 4 Mulago National Specialized Referral Hospital, Kampala, Uganda; 5 National Health Laboratory and Diagnostics Services, Ministry of Health, Uganda

**Keywords:** COVID-19, antibodies, cytokines, clinical chemistry, biomarkers, hematology, viral load (VL) RNA copy numbers, SARS-CoV-2

## Abstract

There are many uncertainties on the future management of the coronavirus disease 19 (COVID-19) in Africa. By July 2021, Africa had lagged behind the rest of the world in Covid-19 vaccines uptake, accounting for just 1.6% of doses administered globally. During that time COVID 19 was causing an average death rate of 2.6% in Africa, surpassing the then global average of 2.2%. There were no clear therapeutic guidelines, yet inappropriate and unnecessary treatments may have led to unwanted adverse events such as worsening of hyperglycemia and precipitating of ketoacidosis in administration of steroid therapy.

in order to provide evidence-based policy guidelines, we examined peer-reviewed published articles in PubMed on COVID 19, or up-to date data, we focused our search on publications from 1st May 2020 to 15th July, 2021. For each of the studies, we extracted data on pathophysiology, selected clinical chemistry and immunological tests, clinical staging and treatment.

Our review reports a gross unmet need for vaccination, inadequate laboratory capacity for immunological tests and the assessment of individual immune status, clinical staging and prediction of disease severity.

We recommend selected laboratory tools in the assessment of individual immune status, prediction of disease severity and determination of the exact timing for suitable therapy, especially in individuals with co-morbidities.

## Introduction

The coronavirus disease 19 (COVID-19) is a highly transmittable and pathogenic respiratory viral infection caused by severe acute respiratory syndrome coronavirus 2 (SARS-CoV-2)^1^. It emerged in Wuhan, China at the end of 2019 and spread so fast around the world, such that by the end of June 2021, it had caused 182 million infections and 3.9 million deaths worldwide^2^. During this time, most African countries experienced fewer infections in the first and second waves but had a terrible third wave, owing to the new Delta variant that eventually accounted for over 80% of the new infections on the continent, by mid-2021. As of 15th July 2021, Africa had registered 4,495,778 infections and 109,359 deaths^3^ at an average death rate of 2.6%, surpassing the global average of 2.2% 2.

By July 2021, there were several SARS-CoV-2 variants of pango lineages including B.1.1.7 (Alpha), B.1.351 (Beta), B.1.617.2 (Delta), P.1 (Gamma), B.1.526 (Iota), B.1.427/B.1.429 (Epsilon) B.1.525 (Eta), P.2 (Zeta), C.37 (lambda) and some combinations^4^,^5^. The delta and lambda variants wee producing a severe form of the disease and contributing to high morbidity and mortality in different countries^6^.

With the discovery of covid-19 vaccine, and considering that many countries in Europe and USA had reached a target vaccination of 70% by June 2021, the global fatality rate started falling and most lockdowns were eased^7^,^8^. However, Africa had lagged behind the rest of the world in Covid-19 vaccinations, accounting for just 1.6% of doses administered globally. In June 2021, the most populous countries on the continent, vaccination rates remained at only 1.9 doses of vaccines administered per 100 people in Nigeria, 1.8 doses in Ethiopia, 2.6 doses in Kenya and 2.2 in Uganda^9^. Regardless of the vaccination coverage, studies showed that previously infected and or vaccinated individuals might not be equally protected against all SARS-CoV-2 variants^10^, casting doubt on the future management of the pandemic. There is an urgent need to monitor the development of the cell-mediated and humoral-mediated immune responses as well as the effectiveness of using neutralizing antibodies in the population.

COVID 19 therapeutic management presented a dual challenge^11^,^12^. The pathophysiology of disease severity was associated with increase in inflammatory cytokines in peripheral blood^13^. For the management of this cytokine “shower”^14^, there is need to build immunity with, probably, immune boosters. In severe disease, the cytokine “shower” turns into a cytokine “storm”. At this point, there is need to suppress the exaggerated immune response with steroids, such as dexamethasone.^15^,^16^. It is thought that there is a narrow window, during this cascade, when administration of steroids can actually prevent the storm^17^. However, the actual timing of that switch, when immune boosters should be replaced with immune suppressors, has not been correctly described. As such, therapies for COVID 19 were used without evidence of benefit and without regard to timing of the disease process^18^. This could potentially cause harm especially among patients who may have had comorbidities^19^,^20^. Steroid therapy, for one, has a bidirectional relationship in their use. Data regarding the usefulness of dexamethasone therapy is encouraging but, when given unnecessarily early, is likely to lead to worsening of hyperglycemia and precipitating ketoacidosis and hence the COVID-19 disease^21^,^22^. It is important to provide clinicians with laboratory-based evidence to make the decision on when the advantages of administration of dexamethasone outweighs its side effects.

The staging of COVID 19 clinical phase is, therefore, critical in the appropriate timing of therapeutics. This study takes a leaf from the management of patients with HIV/AIDS where disease severity determination and the correct timing of interventions has been critical in the management of HIV/AIDS. Griffin et al^23^, described eight clinical stages of COVID-19. These stages include the pre-exposure phase, the incubation period, the detectable viral replication phase, the viral symptom phase, the early inflammatory phase, the secondary infection phase, the multisystem inflammatory phase and the tail phase. We correlated this staging with that of the World Health Organization (WHO) 24. To accurately pinpoints these stages, the laboratory has to take lead.

Laboratory parameters such as a higher prevalence of detectable SARS-CoV-2 plasma viral load 25, lower absolute lymphocyte counts and increased markers of inflammation, such as C-reactive protein and IL-6, are associated with worse respiratory disease severity and an increased risk of mortality 13. It is envisaged, that these laboratory parameters, once monitored regularly, may aid in the risk stratification of patients with COVID19 and early instituting of the appropriate treatment tailored to the stage of the disease progression.

In this report, the review evaluated the available literature to describe the clinical and laboratory staging parameters that may be used in Africa and beyond to determine individual immune response status, predict disease severity, determine the exact timing of suitable therapy, prevent unwanted therapeutic adverse event and subsequently monitor disease prognosis.

## Methods

We examined peer-reviewed published articles in PubMed published in the period spanning the 1^st^ of May 2020 to 15^th^ July 2022. We followed the endorsement of the Preferred Reporting Items for Systematic Reviews and Meta-Analyses (PRISMA). The primary goal of our study was to describe new technologies and the measurement of selected laboratory parameters in order to monitor disease prognosis of COVID 19 in Africa. We used key Boolean search terms with their associated database strings.

### Study Identification and Selection Procedure

The following Boolean search terms with their associated database strings were used to identify literatures on Staging of COVID 19 disease to support clinical and public health interventions in Africa: binding antibodies (IgG, IgA, IgM), neutralizing antibodies (NAbs), Interleukin (IL)-6, Ferritin, Lactate Dehydrogenase (LDH), D-dimer, C-Reactive Protein (CRP) Lymphocyte Count Lymphocyte/Neutrophil Ratio, Viral Load (VL) RNA copy numbers, immune modulators, steroid therapy and support therapy, in relation to prevention and management of severe SARS-CoV-2 infection.

To obtain practical information on the clinical chemistry parameters, immunological tests, clinical staging of the disease and treatment options of COVID 19, we restricted our search to only those records published after the 1^st^ of May 2020 and up to 15 July 2021. The search yielded 4337 records. After removal of the duplicates, 399 records were obtained from which the reviews, preprints, studies with unclear data were removed to obtain 122 records. These were screened for abstract, tittle and overall methodological quality to obtain 75 records for inclusion in the systematic review.

### Findings

We identified 4337 studies; 1412 on clinical chemistry parameters, 1362 on immunological tests; 419 on clinical staging and 1144 on treatment. From these, 3938 were duplicates leaving 399 records eligible for screening. Of these, we excluded the pre-prints and reviews leaving 122 records that met the inclusion criterion. From these 47 studies were excluded with specific reasons, leaving 75 studies for final evaluation and review. (Figure 1).

**Figure:**
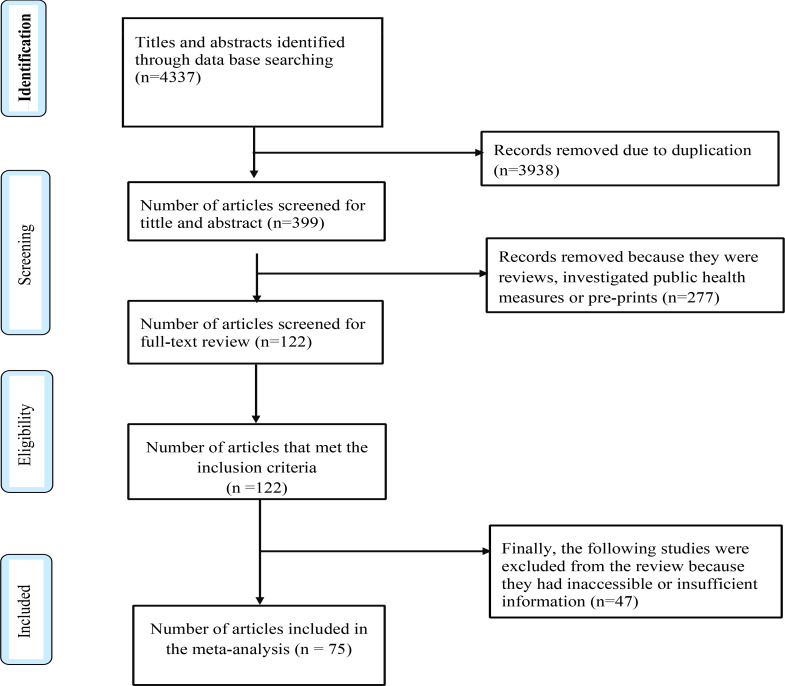


### COVID 19 clinical disease and management of comorbidities

In general, COVID 19 was seen most often in adult male patients between 34 and 59 years, while the highest proportion of severe cases of the disease occurred in adults ≥60 years of age 26, 27. Additional poor prognostic epidemiological risk factors to the disease included smoking and associated comorbidities such as obesity, hypertension, diabetes mellitus, chronic obstructive pulmonary disease, asthma, hypertension, other cardiovascular disease and chronic kidney disease 26, 27. Over the course of COVID 19, patients become depressed and their cortisol levels get elevated through activation of hypothalamic-pituitary- adrenal axis (HPA)^28^,^29^. COVID-19 patients experience/span>polypharmacy^30^ including herbal preparations without approved activity which may lead to drug-drug interactions. The more the number of risk factors, the more was the severity of the disease at presentation^31^,^32^, ^33^, ^34^. Severe manifestations may also be also associated with co-infections of bacteria, malaria and fungi^35^,^36^. These may further worsen the condition, complicating the disease process and disrupting the clinical staging.

COVID 19 in Africa was coupled with the usual phenomenon of colliding epidemics of infections (HIV, TB, Malaria), plus the relatively recent chronic Non-Communicable Disease (NCD) co-morbidities, against the back drop of poor clinical and diagnostic services^37^. Management of the multiple comorbidities can be integrated to help patients, but relatively few health facilities in Africa have focused on screening and linking people suffering from COVID 19 and other multiple illnesses^38^. So, the affected individuals do not frequently receive specialized treatment that addresses all their problems. African health facilities need support to address the gaps in diagnostic services to effectively make proper and prompt decisions and to effectively manage disease outcomes especially in COVID 19 and its exacerbating co-morbidities.

### The changing clinical picture in Africa

In the first wave, March -July 2020, none of the African countries appeared in the list of those with high levels of infection^39^. Apart from the few countries like South Africa, Egypt, Morocco and Nigeria where some patients deteriorated to severity, in most cases, individuals were either asymptomatic or with mild form of the disease. This caused a debate on the infection dynamics in Africa^40^. It is possible that cases were missed due to a lack of adequate detection methods or diagnostic tools, but several other factors could account for the low case numbers. The population was much younger than in Europe, North America and China^41^ plus the generally low population density and low urban population in Africa. But some other scientists hinted on the low transmission of the virus in the tropics^42^.

However, by July 2021, the Delta variant changed the land scape and accounted for over 80% of the new infections in Africa. The more severely infected individuals were young adults, and the infectivity and mortality rates kept rising^43^,^44^. This meant that careful surveillance remained immensely important to monitor host adaptations, viral evolution, infectivity, transmissibility, and pathogenicity. This prompts a critical analysis of the disease mechanisms in the African setting, formulation of an African specific response and probably (if necessary) avoid the ‘cut-paste’ COVID 19 response policies that Africa has adopted from the west for most of 2020 and early 2021.

### Case definition of COVID 19

The presenting signs and symptoms of COVID-19 vary among individuals. WHO has published several editions of “Clinical management of COVID-19: living guidance”^24^ and in these document the clinical symptoms and signs of COVID 19 were summarized, but briefly; The common symptoms at onset of illness are fever and dry cough. Most individuals experience fever (83–99%), cough (59–82%), fatigue (44–70%), anorexia (40–84%), shortness of breath (31–40%), myalgia (11–35%). Other non-specific symptoms, such as sore throat, nasal congestion, headache, diarrhea, nausea and vomiting, have been reported. Loss of smell (anosmia) or loss of taste (ageusia) preceding the onset of respiratory symptoms has also been reported. Some patients suffer from severe pneumonia, progressing rapidly to Acute Respiratory Stress Syndrome (ARDS) and septic shock, which is eventually followed by multiple organ failure and death in about 10% of patients^45^.

Older people, and immunosuppressed patients in particular, may present with atypical symptoms such as fatigue, reduced alertness, reduced mobility, diarrhea, loss of appetite, delirium and absence of fever^46^,^47^,^48^. Symptoms such as dyspnea, fever, gastrointestinal (GI) symptoms or fatigue due to physiologic adaptations in pregnant women, adverse pregnancy events, or other diseases such as malaria, may overlap with symptoms of COVID- 19^49^,^50^.

### Vaccination and immunity

By 16^th^ July 2021, a total of 3,572,787,631 people had been vaccinated across the globe, accounting for 45.8 doses per 100 people^51^. Africa had received only 1.6% of these vaccine doses^9^. It is not clear how long the COVID-19 vaccines will protect people, but Pfizer and BioNTech reported in June 2021 that immunity from their RNA vaccine was still strong (91.3% effective) six months after the second dose^52^.

Natural immunity (i.e., immunity in people who have been infected with COVID-19) can last for up to eight months^53^. However, some studies have showed that previously infected and or vaccinated individuals might not develop immunity and if they do, they may not be equally protected against all SARS-CoV-2 lineages^10^. In-fact some previously infected individuals may be more susceptible to reinfection from the delta variant^54^. In one study, although 88% of people still had antibodies that could block SARS-CoV-2 infection of cultured cells with the Wuhan variant, after 12 months, only 50% of these individuals had antibodies that could block the delta variant^55^.

It is also noteworthy that the strength of the immune response and the time the immunity lasts are very variable (56). Up to 9% of infected people may not have detectable antibodies and about 7% may not have T cells that recognize the virus after 30 days of infection. Up to 5% of people may lose their immune protection within a few weeks^56^. Some people have had repeat COVID 19 infections within a month and although rare, severe disease and death have been recorded among these patients^57^. This complicates the immune pathogenesis during COVID 19 infection and limits our understanding of its management. All these necessitates the continued monitoring of these immune response parameters in the laboratory together with the clinical signs and symptoms as a way of prevention and management of these COVID-19 cases both clinically and in the suspected cases in the communities.

### Laboratory parameters used in monitoring of severely sick COVID-19 patients

The current COVID 19 diagnostic standard combines clinical symptoms with molecular diagnostics (real time PCR) to detect for the presence of viral antigens^58^. There are other various laboratory parameters that have been devised in the routine monitoring of the COVID-19 disease, but no systematic approach has been adapted to follow disease progression from asymptomatic, to mild and severe form of the disease.

We present alternative available parameters that may be used in the monitoring of COVID 19 patients to include the following:

### Neutralizing and Binding antibodies

The persistence of neutralizing antibody (NAbs) responses to COVID 19, among previously infected or vaccinated individuals, is a critical step for patient survival. There was a correlation between early presence of NAbs, say within the first 14 days of symptoms onset, with the time to a negative swab result. On the other hand, compromised immune responses to the SARS-CoV-2 spike was a major trait of COVID-19 patients with critical conditions^62^ and the lack of neutralizing capacity correlated with an increased risk of a fatal outcome^63^.

Until recently, NAbs were identified using the conventional virus neutralization test (cVNT)^64^ or the pseudovirus-based VNT (pVNT), which detect neutralizing antibodies (NAbs) in a patient's blood^65^. The cVNT requires handling live SARS-CoV-2 in a specialized biosafety level 3 (BSL3) containment laboratory facility. It is tedious and time-consuming, taking 2–4 days to complete. The pVNT on the other hand, can be performed in a BSL2 laboratory facilities, but still requires the use of live viruses and cells.

A surrogate VNT (sVNT) that detects NAbs, without the need for any live virus or cells was recently described. The test uses purified receptor-binding domain (RBD) from the S protein and the host cell receptor for Angiotensin Converting Enzyme-2 (ACE2) that mimicks the virus-host interaction in an enzyme-linked immunosorbent assay (ELISA) plate well. In this test, the RBD-ACE2 interaction is neutralized by specific NAbs in patient sera in the same manner as in cVNT or pVNT^66^. The assay can be completed in 1–2hrs in a BSL2 laboratory facility. Total binding antibodies (BAbs) can be determined using ELISA and lateral flow assay (LFA) rapid tests^67^. Immunoglobulin G (IgG) to spike antigens provides the best correlate of neutralization^62^. Both NAb and binding antibody kits for COVID 19 were approved by several agencies.

### Clinical Chemistry

Patients critically ill with COVID-19 feature hyper-inflammation, and the associated biomarkers may be beneficial for risk stratification 68. They have showed metabolic acidosis, blood coagulation dysfunction and prolonged prothrombin time, increase in C - reactive protein (CRP), lactate dehydrogenase (LDH), Aspartate Amino Transferase (AST) enzyme, Troponin, D-dimer, Ferritin and Creatine kinase. However, CRP, D-dimer and Ferritin may be used to track the severity of disease^69^.

### Immune Chemistry

COVID-19 infected patients had a higher plasma level of proinflammatory cytokines including interleukin (IL)-1β, IL-2, IL7, tumor necrosis factor (TNF)-a, interferon (IFN)-γ, IFN-γ-induced protein (IP)10, granulocyte-colony stimulating factor (GSCF) and monocyte chemo-attractant protein (MCP)-1 than healthy adults. Severe and critically ill COVID 19 patients had very high levels of other inflammatory markers, IL-6, IL-4, IL-10, TNF-a. 60 IL-6 may be used to track the severity of disease^70^,^71^.

### Heamogram

In the COVID-19 infected patients, there is leukopenia especially lymphopenia (in 80% of cases)^72^,^73^,^74^, mild thrombocytopenia^75^,^76^ and an increased Erythrocyte sedimentation rate (ESR)^77^,^78^. However, leukocytosis has also been reported 79. Some researchers have suggested that neutrophil-to-lymphocyte ratio (NLR) could ben independent risk factor for severe illness and NLR has been considered as threshold for progression to severe illness in COVID-19 patients^80^.

### SARS-CoV-2 Viral Load (RNA copy Numbers)

Quantitative viral load (VL) determination in blood and respiratory samples can help in early identification of patients at higher risk of severe COVID 19^81^.

Plasma viral load testing has been preferred as a means of monitoring antiretroviral treatment response in HIV-infected patients management. The viral load done on Plasma separation cards (PSCs), used in HIV^82^, correlated well with those from EDTA samples. They have adequate sensitivity and specificity and are unaffected by temperature and storage conditions. Similarly, the PSC can be used in the COVID19 analysis and monitoring of the progression and prognosis of the patient.

SARS-CoV-2 load can be quantified by Droplet Digital Polymerase Chain Reaction (ddPCR). The median plasma values of viral load of around 600 (209–1023) copies/mL corresponded to about 9,404,000 [586,060-10,000,000] copies /mL in respiratory tract samples 81. SARS-CoV-2 viremia is associated with higher transmission^83^, higher hematological malignancies, worse respiratory disease severity, increased risk of mortality and can be used to predict prognosis 25.

## Discussions and Conclusions

Severe COVID 19 patients had more laboratory abnormalities compared to those with moderate or mild disease. Efforts to control spread and progression of COVID 19, such as institutional quarantine and isolation measures, as well as appropriate clinical management of patients, require useful screening and diagnostic tools. Since some COVID 19 patients progressed rapidly to acute respiratory distress syndrome (ARDS) and septic shock, followed by multiple organ failure, proper management of COVID 19 should ensure the early recognition of disease, severe disease or the potential for severe disease.

However, several challenges need to be addressed in the management of COVID 19 in Africa; First, very few people in Africa were vaccinated, acquisition of natural immunity cannot be guaranteed and when it occurs, laboratory methods to confirm acquisition of immunity are not well developed. Secondly, COVID 19 therapeutic management presents a dual challenge, before and during the cytokine “shower”, therefore there is need to build immunity with, probably, immune boosters. Once the cytokine shower turns into the cytokine “storm”, there is need to suppress the exaggerated immune response with steroids. But, the actual timing of that point amongst COVID 19 infected individuals, when no-intervention or when immune boosters should be stopped and immune suppressors should start had, hitherto, not been described. Thirdly, un-necessary steroids can turn pre-diabetics into diabetics and cause several other side effects. Their administration should be regulated with good scientific evidence.

Data from this analysis (Table 1) emphasizes the use of immunological, clinical-chemistry, immune-chemistry and hematological parameters to support the early recognition of severe disease or the potential for severe disease in the management of COVID 19. This may not only reduce the loss of life, but also prevent inappropriate treatments and chronic retention in health care facilities. Ultimately, our suggestion to identify specific stages in the disease management may reduce the high out-of-pocket expenditures on COVID 19 care and the loss of incomes. This review has provided evidence of the sophisticated but now importantly emerging tests for immune effectiveness, such as testing for Neutralizing antibodies. The findings established in the review can be used to predict severe COVID 19 diseases, stage the Covid 19 disease progression and prognosis as well as determine when to give/stop useful but potentially dangerous therapies and support the clinicians in monitoring disease progression.

**Table 1:** Clinical and laboratory Classification of COVID 19 severity

Stage by Griffin (WHO Stage)	Aetiology/ pathophysiology/ clinical symptoms and guidelines		Expected laboratory parameters		Ref ranges	Therapeutic strategy/ guidelines
1. Pre-exposure phase	No symptoms especially among the young people Routine Health Measures for Older adults Diabetics Hypertensive Asthmatics Smoking Cessation	a.	IL-6 (pg/ml)	Normal	0–16.4	**Prophylaxis** Vitamin D Zinc Vitamin C **Immunization** Vaccination Passive Immunization Monoclonal antibodies
		b.	D-dimer (mg/L)	Normal	0–500	
		c.	CRP (mg/L)	Normal	0–1	
		d	LDH (U/L)	Normal	100–250	
		e	Ferritin (ug/L)	Normal	12–300	
		f	Lymphocyte Count/mL	Normal	1000–4000	
		g	Lymphocyte/Neutrophil Ratio	Normal	0.78–3.58	
		h	Thrombocyte count/ uL	Normal	150–450	
		i	Binding Abs (IgG, IgA, IgM)	-ve	-ve	
		j	Neutralizing Abs	-ve	-ve	
		k	VL (RNA copy Numbers)	0	0	
2. Incubation period	Starts from Time of Exposure (TE), as a result of unprotected encounter 15 minutes (continuous or cumulative) Proximity of < 2 meters Enhanced health measures for Older adults Diabetics Hypertensive Asthmatics Incubation 2–14 days	a.	IL-6 (pg/ml)	Normal	0–16.4	Prophylaxix Immunization remains controversial Blocking Viral Replication Blocking Cellular Entry
		b.	D-dimer (mg/L)	Normal	0–500	
		c.	CRP (mg/L)	Normal	0–1	
		d	LDH (U/L)	Normal	100–250	
		e	Ferritin (ug/L)	Normal	12–300	
		f	Lymphocyte Count/mL	Normal	1000–4000	
		g	Lympocyte/Neutrophil Ratio	Normal	0.78–3.58	
		h	Thrombocyte count/ uL	Normal	150–450	
		i	Binding Abs (IgG, IgA, IgM)	-ve	-ve	
		j	Neutralizing Abs	-ve	-ve	
		k	VL (RNA copy Numbers)	Absolute Value	<1000	
3. Detectable viral replication phase (Asymptomatic)	RNA viral copies reduce to below infectious level by day 10 This phase ends when there is no more shedding A few individuals, however, shed without symptoms Enhanced health measures for Older adults Diabetics Hypertension Asthmatics **Early detection of Neutralizing antibodies with rising titers is associated with good recovery**	a.	IL-6 (pg/ml)	Normal	0–16.4	Monoclonal antibodies Augment immune response Interferons
		b.	D-dimer (mg/L)	Normal	0–500	
		c.	CRP (mg/L)	3.1–17.9	0–1	
		d	LDH (U/L)	250–300	100–250	
		e	Ferritin (ug/L)	Normal	12–300	
		f	Lymphocyte Count/mL	Normal	1000–4000	
		g	Lymphocyte /Neutrophil Ratio	Normal	0.78–3.58	
		h	Thrombocyte count/ uL	Normal	150–450	
		i	Binding Abs (IgG, IgA, IgM)	-ve	+ve	
		j	Neutralizing Abs	-ve	+ve	
		k	VL (RNA copy Numbers)	Absolute Value	1000–100000	
4.Viral Symptom phase (Mild disease)	7–14 days after exposure Symptomatic Patients meeting the case definition for COVID-19 without evidence of viral pneumonia or hypoxia Loss of taste Loss of smell Gastro intestinal presentation Cough Fever Myalgia Influenza-Like illness Strengthened health measures (Physician) for Older adults Diabetics Hypertensive Asthmatics **Detection of Neutralizing antibodies with rising titers is associated with good recovery**	a.	IL-6 (pg/ml)	16.5–20	0–16.4	Antiviral therapies Remdesvir Monoclonal antibodies
		b.	D-dimer (mg/L) 0.5–2.6	0–0.5		
		c.	CRP (mg/L)	3.1–17.9	0–1	
		d	LDH (U/L)	250–300	100–250	
		e	Ferritin (ug/L)	301–450	12–300	
		f	Lymphocyte Count/mL	Normal	1000–4000	
		g	Lymphocyte /Neutrophil Ratio	Normal	0.78–3.58	
		h	Thrombocyte count/ uL	Normal	150–450	
		i	Binding Abs (IgG, IgA, IgM)	+ve	+ve	
		j	Neutralizing Abs	+ve	+ve	
		k	VL (RNA copy Numbers)	Absolute Value	100000–1000000	
5. Early inflammatory phase (Moderate Disease)	Pneumonia Adolescent or adult with clinical signs of pneumonia (fever, cough, dyspnoea, fast breathing) but no signs of severe pneumonia, including SpO2 ≥ 90% on room air Cytokine storm Cardiac dysfunction Renal failure Neurologic Manifestations Multi-organ dysfunction **Detection of Neutralizing antibodies is associated with good recovery**	a.	IL-6 (pg/ml)	25–120	0–16.4	Hospitalization Steroids should start now not earlier Non-invasive Ventilation Early intubation is **NOT** recommended Positioning of the patient Anticoagulants
		b.	D-dimer (mg/L)	2.5–3.5	0–0.5	
		c.	CRP (mg/L)	20–140	0–1	
		d	LDH (U/L)	301–350	100–250	
		e	Ferritin (ug/L)	301–700	12–300	
		f	Lymphocyte Count/mL	500–1000	1000–4000	
		g	Lymphocyte /Neutrophil Ratio	>4	0.78–3.58	
		h	Thrombocyte count/ uL	<150	150–450	
		i	Binding Abs (IgG, IgA, IgM)	+ve	+ve	
		j	Neutralizing Abs	+ve	+ve	
		k	VL (RNA copy Numbers)	Absolute Value	100000–1000000	
6. Secondary infection phase (severe Disease)	Severe pneumonia Adolescent or adult with clinical signs of pneumonia (fever, cough, dyspnea, fast breathing) plus one of the following: respiratory rate > 30 breaths/min; severe respiratory distress; or SpO2 < 90% on room air Secondary Infections Need for Culture and Sensitivity	a.	IL-6 (pg/ml)	25–120	0–16.4	High dependence Unit Oxygen therapy Non-invasive Ventilation Additional therapy Antibiotics Antifungal Steroids Anticoagulants
		b.	D-dimer (mg/L)	2.5–3.5	0–0.5	
		c.	CRP (mg/L)	20–140	0–1	
		d	LDH (U/L)	301–350	100–250	
		e	Ferritin (ug/L)	301–700	12–300	
		f	Lymphocyte Count/mL	500–1000	1000–4000	
		g	Lymphocyte /Neutrophil Ratio	>4	0.78–3.58	
		h	Thrombocyte count/ uL	<150	150–450	
		i	Binding Abs (IgG, IgA, IgM)	+ve	+ve	
		j	Neutralizing Abs	+ve	+ve	
		k	VL (RNA copy Numbers)	Absolute Value	100000–1000000	
7. Multisystem inflammatory phase (Critical Disease Sepsis)	Acute life-threatening organ dysfunction caused by a dysregulated host response to suspected or proven infection. Signs of organ dysfunction include: altered mental status, difficult or fast breathing, low oxygen saturation, reduced urine output (3), fast heart rate, weak pulse, cold extremities or low blood pressure, skin mottling, laboratory evidence of coagulopathy, thrombocytopenia, acidosis, high lactate, or hyperbilirubinemia. Peak IgG Secondary Infections	a.	IL-6 (pg/ml)	25–120	0–16.4	Intensive Care Unit Intubation Ventilation
		b.	D-dimer (mg/L)	2.5–3.5	0–0.5	
		c.	CRP (mg/L)	20–140	0–1	
		d	LDH (U/L)	>350	100–250	
		e	Ferritin (ug/L)	301–700	12–300	
		f	Lymphocyte Count/mL	500–1000	1000–4000	
		g	Lymphocyte /Neutrophil Ratio	>4	0.78–3.58	
		h	Thrombocyte count/ uL	<150	150–450	
		i	Binding Abs (IgG, IgA, IgM)	+ve	+ve	
		j	Neutralizing Abs	+ve	+ve	
		k	VL (RNA copy Numbers)	Absolute Value	100000–1000000	
8. Tail phase	Long Haulers Residual symptoms Recurrence of symptoms Subjective/objective manifestations Bimodal pattern Improvement/worsening	a.	IL-6 (pg/ml)	Normal	0–16.4	Subjective treatment
		b.	D-dimer (mg/L)	Normal	0–500	
		c.	CRP (mg/L)	Normal	0–1	
		d	LDH (U/L)	Normal	100–250	
		e	Ferritin (ug/L)	Normal	12–300	
		f	Lymphocyte Count/mL	Normal	1000–4000	
		g	Lymphocyte /Neutrophil Ratio	Normal	0.78–3.58	
		h	Thrombocyte count/ uL	Normal	150–450	
		i	Binding Abs (IgG, IgA, IgM)	+ve	+ve	
		j	Neutralizing Abs	+ve	+ve	
		k	VL (RNA copy Numbers)	0	0	

We conclude that laboratory services to COVID 19 patients should be strengthened so as to provide vital information needed for proper planning and utilization of health resources, in the COVID response and adequately support the effective and efficient delivery of clinical care.

## Recommendations

As noted in the text, some of the poor laboratory prognostic factors include high D-Dimer, lymphopenia, thrombocytopenia, CRP. The review suggests a few additional tests and recommend that the following 10 biomarkers, that have been associated with higher odds of clinical deterioration and death in COVID-19 patients, should be made routine in most African settings. These are;

1. Neutralizing antibodies
2. Binding Abs
  - IgG
  - IgA
  - IgM
3. IL-6
4. D-dimer
5. CRP
6. LDH
7. Ferritin
8. Lymphocyte Count
9. Lymphocyte/Neutrophil Ratio
10. Viral Load (VL) RNA copy Numbers.

It also suggests that centralized laboratories should be supported by African governments to provide Quality assurance services in therapeutics and clinical pharmacology, immunology, clinical chemistry, immunochemistry and hematology to lower-level facilities that conduct these tests.
